# Surgical treatment of Behcet's disease with severe aortic regurgitation

**DOI:** 10.3389/fcvm.2023.1290615

**Published:** 2023-11-20

**Authors:** Chuanbin Tang, Yu Song, Xiaofan Huang, Yuanming Li, Yisilamujiang Tuerxun, Xingjian Hu, Huadong Li, Long Wu

**Affiliations:** ^1^ Department of Cardiovascular Surgery, Union Hospital, Tongji Medical College, Huazhong University of Science and Technology, Wuhan, China; ^2^ Second Affiliated Hospital, Xinjiang Medical University, Urumqi, China

**Keywords:** aortic regurgitation, surgical treatment, Behcet’s disease, diagnosis, medicine therapy

## Abstract

Behcet's disease (BD) is a multisystem inflammatory disease that is characterized by oral aphthosis, genital aphthosis, ocular lesions, and cutaneous lesions. Although BD rarely affects the cardiovascular system, its symptoms can be shown as aortic regurgitation (AR), which requires surgical intervention. Due to the special pathogenesis of BD, a low preoperative diagnosis rate and a high incidence of serious complications, such as perivalvular leakage, valve detachment, and pseudoaneurysm after prosthetic valve replacement, surgical treatment of BD with severe AR has a poor prognosis. In recent years, new surgical strategies have been developed to improve treatment efficacy for this disease. This article reviews and summarizes the evolution of surgical techniques for BD with AR and aims to provide a reference for optimizing surgical strategies, improving perioperative management, and assisting prognosis in patients suffering from BD with severe AR.

## GRAPHICAL ABSTRACT

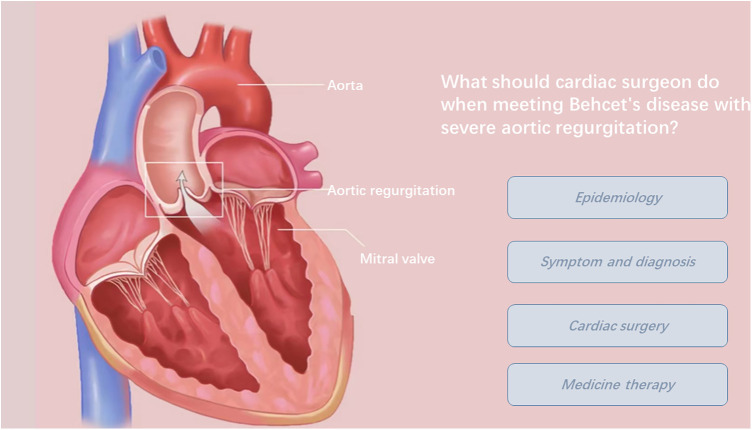

Behcet's disease with severe aortic regurgitation–what we should know.

## Introduction

Behcet's disease (BD) is a chronic vasculitis disease of unknown etiology. The geographic pattern of the disease suggests a distribution along the ancient “Silk Road” with high incidence in the Middle and Far East (1). Turkey has the highest prevalence, affecting 420 people per 100,000 population. In China, the prevalence is 14 people per 100,000 population (1, 2). The mean age of onset is 20–40 years old, and more males are affected than females (3). BD affects multiple systems, including the skin and oral mucosa, eyes, gastrointestinal tract, blood vessels, heart, nerves, bones, and joints (3–5). Because of its high variability of symptoms and lack of specific diagnostic indicators, several diagnostic criteria have been used for identification of the disease, such as International Study Group (ISG) criteria (6), Japan revised criteria (7), Cheng and Zhang criteria (China) (8), and International Criteria for BD (ICBD) (9). Among these, the ICBD formulated in 2014 is most commonly used.

Approximately 1%–6% of patients with BD may have cardiac symptoms, including endocarditis, valve lesions, coronary artery lesions, ventricular thrombosis, pericarditis, myocarditis, and coronary aneurysm (10–12). The Japanese autopsy system suggests that the rate can reach up to 16.5% (13). BD combined with aortic regurgitation (AR) is very common in Chinese and East Asian populations (10, 14).

The cause of its occurrence is BD-induced inflammation, which causes aortic valve leaflet thickening, prolongation, and redundant movements (15, 16). This leads to inadequate and uneven leaflet matching. Another factor is that the thinning of the valve and aortic tissues increases the probability of aneurysm formation during healing (15, 16). Ruptured or perforated valve aneurysms can lead to severe regurgitation and uncontrolled heart failure (15, 17). In addition, BD lacks specific symptoms and diagnostic indicators. Thus, the preoperative diagnosis rate is low, and there is no appropriate treatment in time. These factors result in poor prognosis in patients suffering from BD with severe AR. Early diagnosis and proper treatment for this disease is a major challenge for cardiac surgeons.

Aortic valve replacement is a standard treatment for AR. However, it is difficult for fragile tissue to support high suture tension because BD-induced inflammation affects the aortic valve and aortic root tissues. Postoperative complications, such as paravalvular leaks (PVL) and valve dehiscence/detachment, can easily occur and require two or more cardiac surgeries, and the incidence is 40%–86.7% (18, 19). It is a hot topic to find the best surgical treatment strategy and drug intervention for BD with AR. This paper reviews and summarizes advances in recent surgical methods and medical treatment and aims to provide a reference for reducing surgical complications and improving the prognosis of patients with BD.

## Clinical symptoms and diagnosis of BD with AR

Patients suffering from BD with AR can show non-specific symptoms, such as chest tightness, shortness of breath after activity, chest pain, palpitations, and even acute heart failure (20–22). Lacking specific clinical symptoms and laboratory indicators, the rate of misdiagnosis or missed diagnosis remains high. A variety of diagnostic criteria for BD have been published over the past few decades (6–9). Among these, the ICBD diagnostic criterion proposed by the International Behcet's Disease Research Group in 2014 has both high sensitivity and specificity in the early diagnosis of BD (Table 1) (9). Therefore, ICDB criteria have been highly recommended for the diagnosis of BD.

**Table 1:** International criteria for Behcet's disease.

Symptom	Point
Recurrent oral aphthosis	2
Recurrent genital aphthosis	2
Ocular lesions	2
Skin lesions	1
Neurological manifestations	1
Vascular manifestations	1
Positive pathergy test^a^	1

^a^ The primary scoring system does not include pathergy testing; one more point may be assigned if the pathergy test is positive. Scoring ≥4 indicates Behcet's diagnosis.

### Differential diagnosis

BD combined with AR needs to be differentiated from infective endocarditis (IE), degenerative valve heart disease, and rheumatic valvular heart disease (RVHD) (Table 2). BD combined with valve lesions and IE can have similar clinical symptoms and echocardiographic findings, so they can be easily misdiagnosed. Sometimes patients suffering from valve lesions can even have IE (23, 24). Kang et al. (25) and Hattori et al. (26) found that echocardiography in patients with BD combined with AR mimics signs of IE, such as aortic valve vegetations, perivalvular and subannular abscesses, and valve perforation. In this case, C-reactive protein (CRP), erythrocyte sedimentation rate (ESR), blood culture, and histopathological examination can be used to distinguish between BD and IE.

**Table 2:** Differential diagnosis of BD with aortic regurgitation.

Disease	Patient characteristics	Clinical symptoms	Valves affected	Ancillary examination
Behcet's disease combined with valve lesions	Men aged 20–40 years old	① Non-specific manifestations: chest tightness, shortness of breath and even heart failure; ② BD-specific manifestations (Table 1)	Aortic valve	① Echocardiography: cusp prolapse with aneurysmal changes is common. Vegetation-like lesions and perivalvular abscesses may present. ② Indicators of inflammation may elevate, such as CRP and ESR
Infective endocarditis	Any age	① Fever, night sweats, fatigue ② Vascular phenomena: arterial emboli, splenic infarction, mycotic aneurysms, intracranial hemorrhage, and Janeway lesions ③ Immunological phenomena: Osler's nodes, Roth's spots	Mitral valve	① Echocardiography: Valve vegetation is common; valvular regurgitation is the principal sign of leaflet destruction/perforation ② Typical endocarditis organisms are evident on blood cultures
Rheumatic valvular heart disease	Women aged 20–40 years old	① Non-specific manifestations: chest tightness, shortness of breath, fatigue ② History of rheumatic fever, such as carditis, arthritis, chorea, subcutaneous nodules, erythema marginatum	Often involved the mitral valve and aortic valve; Multiple valve lesions may be present	① Echocardiography: includes chordal fusion and shortening, leaflet thickening and retraction, and leaflet calcification. ② Laboratory tests may show high levels of CRP and ESR
Calcific aortic valve disease	Elderly population	Non-specific manifestations: exertional dyspnea, angina, and dizziness/syncope	Aortic valve	① Echocardiography: aortic stenosis is common, with severe valve calcification and thickening with impaired leaflet motion and vast blood flow obstruction. ② Often associated with dyslipidemia

RVHD has a high morbidity in underdeveloped countries and regions, and women seem more likely to suffer from it (27, 28). Acute rheumatic valvulitis is characterized by annular dilatation and chordal elongation, leading to leaflet prolapse. Over time, mitral stenosis, the hallmark of rheumatic heart disease, will develop as a result of commissural fusion (29). Mixed aortic and mitral valve disease occurs more frequently than isolated aortic valve disease in RVHD. Echocardiography findings include chordal fusion and shortening, leaflet thickening and retraction, and leaflet calcification.

Calcific aortic valve disease (CAVD) is the most common valvular heart disease in developed countries and predominantly affects the elderly population (30). CAVD often presents as aortic stenosis (AS). It is a progressive condition that evolves to severe valve calcification and thickening with impaired leaflet motion and vast blood flow obstruction, which are the hallmarks of calcific AS (31).

Cardiac surgeons should be cautious about patients with aortic valve disease. To avoid misdiagnosis and missed diagnosis, complete medical history collection and ancillary examinations and correct etiological diagnosis are important for the treatment of BD (Table 2).

### Ancillary examination

#### Electrocardiogram

There are no specific manifestations. Atrioventricular block, ventricular parasystole, and sinus tachycardia may occur if inflammation involves the cardiac conduction system.

#### Echocardiography

Transthoracic echocardiography (TTE) or transesophageal echocardiography (TEE) can help provide a quick and visual image of the anatomy of the aortic valve and aortic root and assess cardiac function. The most common manifestation of BD combined with AR is cusp prolapse with aneurysmal changes. Echocardiographic findings mimicking IE can also be present, such as vegetation-like lesions and perivalvular abscesses. When the aorta is involved, aortic dilation, aneurysm formation, aortic pseudoaneurysm, and sinus of Valsalva aneurysm may be found (24).

#### Inflammatory indicators

Inflammatory markers, such as ESR and CRP, may show a negative correlation with the event-free period (32) and can help determine the timing of surgery and predict the efficiency of immunosuppression (33).

#### Histopathological examination

This is the gold standard diagnostic criterion. Pathologically, aortic tissue shows endothelial damage, fibrin deposits, mixed inflammation, microabscesses with granulomatous reactions, granulation tissue, and fibrosis (34, 35). Valve tissue can manifest as mixed acute and chronic inflammation, neutrophil and lymphocyte infiltration, and proliferating capillaries (34, 35).

## Surgery therapy

Due to systemic inflammation, patients with BD have a high postoperative complication rate and mortality, and cardiac surgery should be performed cautiously with inflammation control. Mild-to-moderate AR can be treated medically, while cardiac surgery is needed when severe regurgitation affects cardiac function according to the 2021 ESC/EACTS guidelines for the management of valvular heart disease (36).

Aortic valve replacement (AVR) and aortic root replacement (ARR) are standard techniques for severe AR (21, 37), but postoperative complications in patients with BD, such as perivalvular leakage and pseudoaneurysm, remain high, and the long-term prognosis is poor (15, 18, 34, 38). In recent years, with a better understanding of BD pathophysiology, clinical centers have proposed modified surgical strategies (Table 3) and drug treatment regimens (Table 4) in anticipation of improving the prognosis of BD.

**Table 3:** Surgical treatments for BD with AR.

Time	Author	Surgery	Advantages	Limitations
AVR				
2009	Azuma	Subannular ring reinforcement technique	① Holds the aortic annulus between the subannular ring and the prosthetic valve, reducing rupture stress on the aortic annulus ② Avoid coronary anastomosis ③ Lower risk of hemorrhage and lower medical costs than ARR	① Sample size was too small. ② Limited indications make it unclear if this surgery is suitable for patients with predominant valvular lesions other than the sinus of Valsalva aneurysm
2011	Liang	Modified AVR with reinforcement of the aortic wall	① Reinforces the aortic wall, distributing the tension to the felt and the sewing ring instead of the fragile annulus ② The felt serves as banding to prevent further dilation and dissection. ③ Lower risk of hemorrhage and lower medical costs than ARR	① Sample size was too small. ② Limited indications make it unclear if this surgery is suitable for patients with predominant valvular lesions other than the sinus of Valsalva aneurysm
2012	Tang	Supra-annular aortic valve replacement (SAVR)	① Reconstructs the aortic annulus with a pericardial patch, places a prosthetic valve between the native annular and coronary ostium, and reinforces the aortic wall ② Applicable for the reoperation ③ Avoid coronary anastomosis	① Sample size was too small. ② Limited indications make it unknown if this is suitable for cases lacking normal intima tissue between the native annulus and the coronary ostium
2021	Sun	Modified AVR + aortic root reinforcement	① Uses interrupted mattress stitches in a circumferential fashion beneath coronary arteries ostia. Reinforces the aortic wall between the outer cushion and the sewing ring of the artificial valve ② Reinforces the aortic root is essential ③ Avoids coronary anastomosis	① Sample size was too small. ② Limited indications make it unclear if this surgery is suitable for patients with predominant valvular lesions other than the sinus of Valsalva aneurysm
ARR				
2007, 2008	Yoshikawa, Tsunekawa, and Tao	Mini-skirt Bentall surgery	① The stiff sewing ring of the prosthetic valve is not directly attached to the annulus, making the fragile aortic annulus less mechanically stressed. ② Various reports indicate suitability for primary operation or reoperation. ③ Reconstruct the coronary arteries by button technique	① Multicenter studies and long-term follow-up are needed
2009, 2012	Jeong and Ma	Homograft aortic root replacement	① Homografts have good biocompatibility and can obviate the risk of valve dehiscence. ② Eliminate problems with anticoagulation	① Homografts are scarce. ② Inflammation may involve the homograft. ③ Multicenter studies and long-term follow-up are needed
2017	Chen	Modified Bentall valved conduit attached to left ventricular outflow tract	① Left ventricular outflow tract tissue can withstand greater tension than fragile annulus. ② Rescues patients who underwent prosthetic valve detachment after primary standard AVR ③ Reconstructs the coronary arteries by button technique	① Not suitable for the primary surgery for resection of much aortic root tissue. ② It is not clear whether the mitral valve would be affected. ③ Multicenter studies and long-term follow-up are needed
Other surgical techniques				
2010, 2012	Hollander and Ma	Orthotopic heart transplantation	① Saves lives of patients with end-stage heart disease	① Lack of heart donors ② Surgery and postoperative management for patients are complex
2021	Jiang	TAVR	① Interventional surgery with minimally invasive ② Suitable for patients with a high risk of surgery	① Requires a strict grasp of surgical indication ② The safety and efficacy of TAVR surgery for the treatment of BD need further study

**Table 4:** Perioperative immunosuppressive therapy.

Author	Year	Perioperative immunosuppressive therapy regimen
Ahn (39)	2009	Preoperation and postoperation: oral prednisone 1 mg/kg/day and azathioprine 1.5–2.5 mg/kg/day, prednisolone was slowly tapered to reach normal ESR and CRP levels
Liang (33)	2011	Preoperation: oral prednisone (20 mg/day) and thalidomide (50 mg/night) for 2 weeks Postoperation: methylprednisolone (40 mg/day) intravenous drip for 4 consecutive days; after that, oral administration of prednisolone (10 mg/day) and thalidomide (50 mg/day) was begun
Ma (34)	2012	Postoperation: oral prednisone (0.5 mg/kg/day), followed by oral cyclophosphamide (100 mg/day), taken for 10 days every month. Regularly consult a rheumatologist to control inflammation and change the immunosuppressive regimen
Yang (14)	2018	Preoperation and postoperation: oral prednisone (5 mg/kg/day) and thalidomide (50–200 mg/day), adjust the dose of thalidomide based on erythrocyte sedimentation rate and C-reactive protein
Sun (40)	2021	On the basis of glucocorticoids + immunosuppressants, biologic agents are added to rapidly control inflammation and improve prognosis. Biologics are discontinued 2 weeks before surgery, and postoperative patients without infection can start taking the drug at 4 weeks. TNF*α* inhibitors: IFX was administered at a dose of 5 mg/kg intravenously at 0, 2, and 6 weeks, followed by every 8 weeks. ADA was administered at 40 mg subcutaneously every 2 weeks. GOL was injected subcutaneously at a dose of 50 mg every 4 weeks. Interleukin-6-receptor antagonist: TCZ was administered at a dose of 8 mg/kg intravenously every 4 weeks

ADA, adalimumab; GOL, golimumab; IFX, infliximab; TCZ, tocilizumab.

### Aortic valve replacement

Azuma et al. (15) performed a modified AVR termed the “subannular ring reinforcement technique” in three patients. To reduce the rupture stress to the aortic annulus, they used a vertical 2/0 polyester mattress suture in the supra-annular position and simultaneously placed a polyester ring in the subannular position as a reinforcement, which sandwiched the aortic annulus between the subannular ring and the prosthetic valve. Each vertical suture was horizontally mounted. No operative deaths or prosthetic valve detachments occurred after a mean period of 3 ± 1.8 years (range: 1.5–5.1 months). For patients without a sinus of Valsalva aneurysm, this modified AVR is suitable and possesses advantages, such as less resection and suture of tissues, less rupture stress on the aortic annulus, and no coronary anastomosis. However, this surgery may not be applicable in patients who have sinus of Valsalva aneurysms or undergo reoperation.

Liang et al. (33) proposed a modified AVR technique with reinforcement of the aortic root. They placed a 10.0 cm  × 1.0 cm Teflon felt strip beneath the ostia of both coronary arteries to encircle the aortic root. Using continuous mattress stitches, a double-armed 3-0 polypropylene suture line was placed 1.0 cm beneath the ostium of the left coronary artery and passed from outside to inside through the felt strip and the aortic wall, and the prosthetic valve ring was sequentially sewn. Stitches were then passed in a continuous line retrogradely. The suture was tightened, and the aortic wall was sandwiched between the external subannular felt and the sewing ring of the prosthetic valve. By strengthening the aortic wall instead of the diseased annulus, tension is mostly distributed on the felt and the sewing ring. Meanwhile, the felt strip could serve as a banding to prevent further dilatation and dissection. However, the sample size of this study is too small, and the safety and efficacy of this surgery need to be verified by larger studies and a long-term follow-up.

Sun et al. (18) performed modified AVR with reinforcement of the aortic root on five BD patients with AR. Beneath the ostia of both coronary arteries, they used interrupted mattress stitches from the outside in and horizontally through the aortic wall in the same plane below the valve ring in a circumferential fashion. Then, the prosthetic valve was anchored in the aortic wall, and the ring was sequentially sewn. Performing reinforcement on the aortic root is also an essential step. The tension on the aortic annulus and aortic wall is distributed on the same horizontal, which avoids the formation of shear and prevents the annulus from tearing. The outcome showed a good prognosis, and no deaths or complications were observed in these five patients during the follow-up.

Tang et al. (41) proposed supra-annular aortic valve replacement (SAVR) to rescue valve detachment after AVR attributable to BD. In five patients who underwent aortic valve detachment (with a mean interval of 6 months after the first operation), the aortic annulus was reconstructed with a pericardial patch, and then SAVR was performed. The prosthetic valve was positioned between the native annular and coronary ostium. Then, they placed Teflon felt strips on the outside of the aortic wall for reinforcement. By doing so, surgeons could repair the ventriculo-aortic disconnection and aortic root damage and prevent pseudoaneurysm, mitral insufficiency, paravalvular leakage, and valve detachment. In addition, this method obviates the necessity of coronary artery bypass grafting (CABG), which makes surgery more difficult and potentially leads to the development of occlusive disease in the vein graft. No valve detachment or pseudoaneurysm was reported during the average follow-up of 21.8 months. Despite the limitations of the small cohort of patients and short follow-up time, this surgery is applicable for patients with extensive annular destruction after recurrent failure of conventional methods.

In summary, various modified AVR surgeries avoid suturing the valve prosthesis to the fragile aortic annulus and adopt reducing suture or reinforcement techniques, all of which effectively reduce the incidence of valve detachment and pseudoaneurysm. Compared with ARR, modified AVRs preserve the aortic sinus and avoid coronary anastomosis, which simplifies surgical procedures. However, the indications for modified AVRs are limited, and patients with sinus of Valsalva aneurysms are recommended for ARR. Meanwhile, current reported studies are limited by small sample sizes and short follow-up times, and the safety and long-term effects of modified AVRs require further clinical studies.

### Aortic root replacement

ARR, such as the Bentall operation, which uses artificial or biological materials to replace diseased aortic root tissue, is often used for the treatment of aortic aneurysm/dissection. In recent years, studies have proposed that ARR is better than AVR for the treatment of BD with AR, and homograft grafts are preferred over valved conduits with a mechanical or a bioprosthetic valve (32, 34).

The probability of valve detachment or paravalvular leakage after AVR in patients with BD is 40%–86.7% (18, 19, 32, 34). In the report of Jeong et al. (32), 78.9% of patients needed a reoperation, and 36.8% of patients needed two or more reoperations. The reoperation rate in the AVR group reached 92.3% (12/13). However, the ARR group was only 25% (1/4). The mean event-free survival at 13 years was 39.2% ± 14.1% in patients who underwent a Bentall-type operation, but it was 4% ± 3.9% in patients who had AVR. In the study of Ma et al. (34), seven patients underwent isolated AVR as the primary procedure. Valve dehiscence occurred in six patients (85.7%) postoperatively after a mean interval of 2.9 ± 1.7 months. However, no valve dehiscence occurred in three patients who underwent an ARR. Therefore, ARR seems to be a better choice for BD patients, regardless of primary operation or reoperation.

Yoshikawa et al. (42) used a mini-skirt Bentall procedure to treat a BD patient with an aneurysm of the sinus of Valsalva, dilatation of the annulus, and severe aortic regurgitation. This modified Bentall technique used a composite valved graft in which the aortic valve prosthesis was sutured 1 cm above the proximal end of the graft (43) and then implanted into the aortic valve annulus with pledgeted everting mattress sutures. They reconstructed the coronary arteries by the button technique. No valve detachment or pseudoaneurysm formation was observed 40 months after surgery. Liang et al. (44) reported that eight patients received the mini-skirt Bentall procedure, and no complications were observed in any of the patients during the follow-up. All cardiac function of patients was good. This mini-skirt technique, in which a prosthetic valve ring is not sewn directly to the annulus, makes the fragile aortic annulus less mechanically stressed compared with isolated AVR or standard ARR (45). Thus, it is suitable for either primary operation or reoperation. In addition, it can reduce the incidence of postoperative perivalvular leakage, prosthetic valve detachment, and bleeding risk, avoid coronary anastomotic leakage and pseudoaneurysm, and improve long-term survival. Nevertheless, multicenter studies and long-term follow-ups are still needed to clarify the efficacy and possible problems of this procedure.

BD-induced inflammatory changes can affect both the aortic annulus and the ascending aortic wall, resulting in postoperative valve detachment. Fortunately, left ventricular outflow tract myocardial tissue is free of inflammation (46). Chen et al. (46) designed a modified Bentall procedure: a valved conduit directly sutured to the left ventricular outflow tract instead of the fragile annulus. They reconstructed the coronary arteries by the button technique in five patients with prosthetic valve detachment. All patients who received this procedure after standard AVR had satisfactory short-term and mid-term results. This procedure can be applied to patients who undergo prosthetic valve detachment after primary standard AVR. However, it is not suitable for primary surgery for resecting aortic root tissue. Whether the placement of the valved conduit deep into the left ventricular outflow tract will affect the function of the mitral valve is unknown, and long-term efficacy follow-up is also needed.

### Transcatheter AVR

Jiang et al. (47) reported a 60-year-old patient with severe AR who had been diagnosed with BD for 30 years and had a long history of taking corticosteroids and immunosuppressants. After evaluating the risk of prosthetic valve detachment, infection, and poor wound healing after surgery, they chose transcatheter AVR (TAVR) surgery using a 29-mm JValve valve fixed 1 cm below the aortic annulus. Methylprednisolone and thalidomide were taken to control inflammation after surgery, no complications occurred, and cardiac function improved during 2 years of follow-up.

TAVR is a suitable treatment for inoperable or increased surgical risk patients with severe aortic stenosis (48)^,^ and the majority of currently available transcatheter devices are designed for treating calcified aortic stenosis. Recently, many studies have demonstrated acceptable clinical outcomes of TAVR in patients with AR (49, 50). Adverse events related to TAVR include stroke, PVL, conduction disturbances, coronary occlusion, and reaccess (51). Most patients with BD are young and often have aortic root lesions; thus, open surgery seems more suitable than TAVR. Second, it is a challenge to anchor the prosthesis to the dilated aortic annulus with minimal calcification, especially in BD patients. Therefore, TAVR requires strict surgical indications. During follow-up, Jiang et al. (47) found that aortic valve regurgitation gradually reoccurred, which may be attributed to biovalvular decay or inflammation. Therefore, the safety and efficacy of TAVR surgery for the treatment of BD with AR need further investigation and long-term follow-up.

### Orthotopic heart transplantation

Orthotopic heart transplantation is the gold standard treatment for patients suffering from advanced heart failure (52, 53). Some patients who do not respond well to immunosuppressive therapy (IST) still experience various complications and eventually progress to end-stage heart disease after multiple surgeries. Ma et al. (34) and Hollander et al. (54) reported heart transplantation in these patients, and the postoperative survival time was more than 2 years. Generally, heart transplantation is considered to be contraindicated in BD due to the risk of performing the needed vascular and atrial anastomoses in the presence of severe inflammation (53). Moreover, patients may develop other severe systemic diseases after surgery, such as kidney failure and lung infections. Due to the lack of clinical studies with large sample sizes, the benefits and efficacy of heart transplantation in BD patients remain unclear. For BD patients who have progressed to the end-stage stage, heart transplantation can be the ultimate alternative. However, various postoperative complications and complex drug treatments may be a challenge for clinicians. IST is important. The interleukin receptor antagonist IL-1RA has been used to prevent associated vascular disease, but the risks associated with IL-1RA in heart transplant patients are unclear.

## Medicine therapy

There is no strong evidence of medical therapy for the treatment of AR in BD thus far. The main purpose of medical therapy is to inhibit the progression and recurrence of inflammation and prevent irreversible damage. Drugs used for treatment included glucocorticoids (GCs), immunosuppressants, and biologics (Table 4). Many studies have demonstrated that the usage of GCs in conjunction with immunosuppressants could effectively decrease the development of PVL and other complications (34, 55). However, using GCs and immunosuppressants during surgical treatment could delay wound healing and increase susceptibility to infection. Some investigations suggest that biological agents can reduce the dose and side effects of GCs and immunosuppressants and improve prognosis (40). Commonly used immunosuppressants include cyclophosphamide, thalidomide, and azathioprine (45). Biologic agents include TNF-α antagonists and IL-6 antagonists (40, 56). Perioperative medicine regimens vary in the literature and should be individualized for the patient.

## Timing of IST

It remains controversial when to start IST. Some investigations have suggested that early detection of BD and starting an effective IST before the first operation might provide the best clinical outcomes (55). However, some researchers argue that postoperative, but not preoperative, IST is an independent predictor of less development of PVL (57). There was no significant difference in the occurrence of reoperation or death between preoperative and postoperative IST vs. postoperative IST only (38). This means that starting IST after surgery plays a more essential role in reducing postoperative complications. Inflammatory markers such as ESR and CRP, which could reflect BD activity, are proposed to help determine the timing of surgery and predict the effectiveness of IST (32, 40, 55). Keeping the ESR under 32.5 mm/h before the operation resulted in better outcomes (55). In our opinion, patients who will receive elective surgery with inactive inflammation are recommended for both preoperative and postoperative IST. For patients who will receive emergency surgeries with medically intractable severe AR and congestive heart failure regardless of active or inactive inflammation, IST should be started as soon as possible postoperatively.

## Postoperative follow-up

GCs, immunosuppressants, and anticoagulants should be taken for a long time after surgery. The patients had regular postoperative reviews in the departments of cardiac surgery, rheumatology, and immunology. The dosage of medicine should be adjusted according to symptoms and test results, such as ESR, CRP, and coagulation function. TTE should be routinely examined every 3–6 months. TEE and total aortic computed tomography angiography (CTA) should be performed when complications, such as perivalvular leakage and prosthetic valve avulsion, are suspected.

## Summary

In China and East Asia, BD with valvular heart lesions often presents with aortic regurgitation. Despite the low prevalence, BD with AR has a lower preoperative diagnosis rate, a higher rate of postoperative complications, and a poorer prognosis than AR caused by other factors. The diagnosis of BD is mainly based on ICBD diagnostic criteria, and patients lack specificity in their clinical symptoms, which makes the accurate diagnosis of BD a great challenge for cardiac surgeons. For patients with a suspected or definitive diagnosis of BD, a multidisciplinary team composed of cardiac surgery, rheumatology and immunology, infectious diseases, and pathologists shall jointly decide the treatment plan to improve the preoperative diagnosis rate and the prognosis of BD.

The prognosis of BD with AR is related to the surgical technique. Isolated AVR is not suitable for the surgical treatment of such patients. Cardiac surgeons should select the appropriate surgical technique according to the condition of the patient. For patients without aneurysms of the Valsalva sinus, various modified AVRs or mini-skirt Bentall surgeries can be selected in combination with the primary surgery. If an aneurysm of the Valsalva sinus is present, ARR surgery should be performed, such as the mini-skirt Bentall surgery. The Button technique is recommended for the reconstruction of the coronary arteries. If patients undergo reoperation when valve detachment or pseudoaneurysm appears, supra-annular AVR or various modified Bentall procedures can be selected. For patients with great surgical risk, interventional surgery (TAVR) may be an option. For patients with severe complications and end-stage heart failure despite multiple modified surgeries, heart transplantation can be applied.

**Figure 1:**
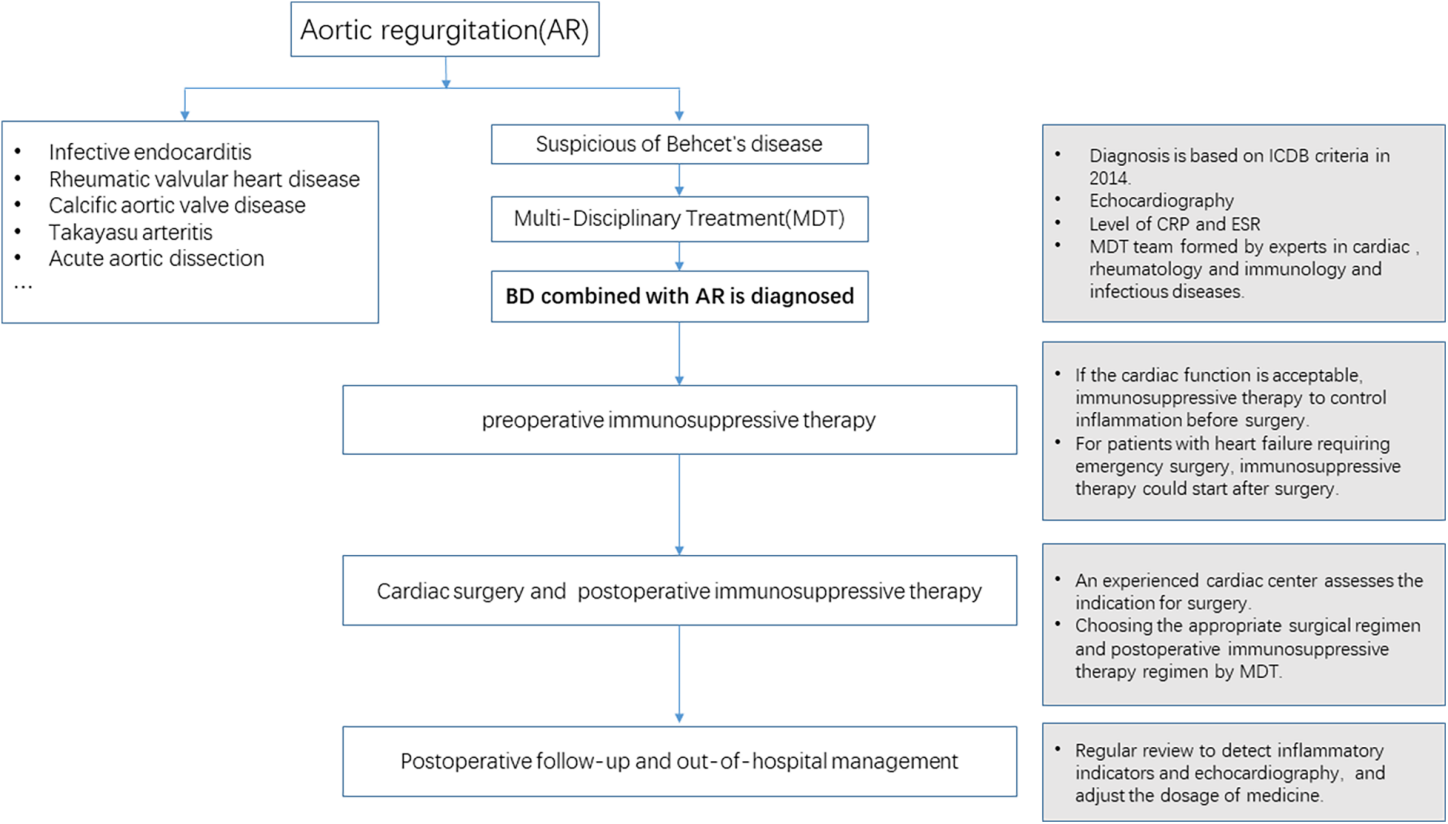
Flowchart for the diagnosis and treatment of BD combined with AR.

Perioperative treatment with GCs and immunosuppressants is essential. Inflammation should be controlled within normal levels before cardiac surgery. Biologics can be used if necessary. Because patients have a critical condition and require emergency surgery but do not receive sufficient anti-inflammatory therapy before surgery, they should start treatment with GCs and immunosuppressants immediately after surgery. Perioperative inflammatory indicators, such as CRP and ESR, should be monitored, and echocardiography should be reviewed regularly to closely monitor the condition of the patient (Figure 1).

Although many studies and surgical techniques have been proposed for the treatment of BD, these studies are limited by small sample sizes and short follow-up times. Thus, there is currently no consensus on the treatment strategy and long-term effect of BD. Multicenter, prospective, randomized controlled large-sample clinical trials on this disease are needed.

## References

[1] D’Alessandro S Nicolini F Formica F. Commentary: a novel surgical strategy for aortic valve replacement in Behcet's disease: is this the new Silk Road? JTCVS Tech. (2020) 2:48–9. 10.1016/j.xjtc.2020.03.010 34317748 PMC8298920

[2] Ghang B Kim JB Jung SH Chung CH Lee JW Song JM Surgical outcomes in Behcet’s disease patients with severe aortic regurgitation. Ann Thorac Surg. (2019) 107(4):1188–94. 10.1016/j.athoracsur.2018.08.040 30315793

[3] Davatchi F Chams-Davatchi C Shams H Shahram F Nadji A Akhlaghi M Behcet’s disease: epidemiology, clinical manifestations, and diagnosis. Expert Rev Clin Immunol. (2017) 13(1):57–65. 10.1080/1744666X.2016.1205486 27351485

[4] Chen J Yao X. A contemporary review of Behcet’s syndrome. Clin Rev Allergy Immunol. (2021) 61(3):363–76. 10.1007/s12016-021-08864-3 34076835

[5] Fei Y Li X Lin S Song X Wu Q Zhu Y Major vascular involvement in Behcet’s disease: a retrospective study of 796 patients. Clin Rheumatol. (2013) 32(6):845–52. 10.1007/s10067-013-2205-7 23443336

[6] Criteria for diagnosis of Behcet’s disease. International study group for Behcet’s disease. Lancet. (1990) 335(8697):1078–80. 1970380

[7] Mizushima Y. Recent research into Behcet’s disease in Japan. Int J Tissue React. (1988) 10(2):59–65. 3053482

[8] Chen SP. Some special clinical manifestations of Behcet’s disease—report of illustrative cases and review of literature (author’s transl). Zhonghua Nei Ke Za Zhi. (1980) 19(1):15–22. 7472023

[9] International Team for the Revision of the International Criteria for Behcet’s Disease (ITR-ICBD). The International Criteria for Behcet’s Disease (ICBD): a collaborative study of 27 countries on the sensitivity and specificity of the new criteria. J Eur Acad Dermatol Venereol. (2014) 28(3):338–47. 10.1111/jdv.12107 23441863

[10] Xuedong Z Jin W Guangfa Z. Clinical study of Behcet’s disease with cardiac valve involvement. J Cardiovasc Pulmon Dis. (2017) 36(11):905–8.

[11] Cocco G Gasparyan AY. Behcet’s disease: an insight from a cardiologist’s point of view. Open Cardiovasc Med J. (2010) 4:63–70. 10.2174/1874192401004020063 20360978 PMC2847211

[12] Geri G Wechsler B Thi Huong DL Isnard R Piette JC Amoura Z Spectrum of cardiac lesions in Behcet disease: a series of 52 patients and review of the literature. Medicine (Baltimore). (2012) 91(1):25–34. 10.1097/MD.0b013e3182428f49 22198500

[13] Lakhanpal S Tani K Lie JT Katoh K Ishigatsubo Y Ohokubo T. Pathologic features of Behcet’s syndrome: a review of Japanese autopsy registry data. Hum Pathol. (1985) 16(8):790–5. 10.1016/S0046-8177(85)80250-1 4018777

[14] Zongda Y Jiatao F Jingfeng J Yanling S Huaan Y Jiawang L Treatment of aortic regurgitation caused by Behcet’s disease. South China J Cardiovasc Dis. (2018) 24(06):683–7.

[15] Azuma T Yamazaki K Saito S Kurosawa H. Aortic valve replacement in Behcet’s disease: surgical modification to prevent valve detachment. Eur J Cardiothorac Surg. (2009) 36(4):771–2. 10.1016/j.ejcts.2009.05.031 19699654

[16] Grygiel-Gorniak B Oduah MT Olagunju A Klokner M. Disorders of the aorta and aortic valve in connective tissue diseases. Curr Cardiol Rep. (2020) 22(8):70. 10.1007/s11886-020-01314-0 32562158 PMC7305067

[17] Chikamori T Doi YL Yonezawa Y Takata J Kawamura M Ozawa T. Aortic regurgitation secondary to Behcet’s disease. A case report and review of the literature. Eur Heart J. (1990) 11(6):572–6. 10.1093/oxfordjournals.eurheartj.a059752 2190836

[18] Sun X Yuan L Liu J Yang Q Liu H Zhang H The surgical outcomes of aortic valve replacement in patients with aortic valve lesions caused by Behcet’s disease: lessons we learned. Ann Transl Med. (2021) 9(20):1607. 10.21037/atm-21-5673 34790813 PMC8576658

[19] Ando M Kosakai Y Okita Y Nakano K Kitamura S. Surgical treatment of Behçet’s disease involving aortic regurgitation. Ann Thorac Surg. (1999) 68(6):2136–40. 10.1016/S0003-4975(99)00847-4 10616990

[20] Gao N Han W Ci WP Liao H Du J. Clinical data analysis of cardiovascular involvement in Behcet’s disease. Zhonghua Yi Xue Za Zhi. (2016) 96(19):1523–6. 10.3760/cma.j.issn.0376-2491.2016.19.013 27266500

[21] Akinseye OA Pathak A Ibebuogu UN. Aortic valve regurgitation: a comprehensive review. Curr Probl Cardiol. (2018) 43(8):315–34. 10.1016/j.cpcardiol.2017.10.004 29174586

[22] Maurer G. Aortic regurgitation. Heart. (2006) 92(7):994–1000. 10.1136/hrt.2004.042614 16775114 PMC1860728

[23] Jiang X Liu J Khan F Tang R Zhang Y Gu T. Aortic and mitral valve surgery for infective endocarditis with reconstruction of the intervalvular fibrous body: an analysis of clinical outcomes. J Thorac Dis. (2020) 12(4):1427–36. 10.21037/jtd.2020.03.04 32395280 PMC7212136

[24] Pu L Li R Xie J Yang Y Liu G Wang Y Characteristic echocardiographic manifestations of Behcet’s disease. Ultrasound Med Biol. (2018) 44(4):825–30. 10.1016/j.ultrasmedbio.2017.12.010 29373154

[25] Kang HM Kim GB Jang WS Kwon BS Bae EJ Noh CI An adolescent with aortic regurgitation caused by Behcet’s disease mimicking endocarditis. Ann Thorac Surg. (2013) 95(6):e147–9. 10.1016/j.athoracsur.2012.11.027 23706466

[26] Hattori K Tabata M Nojiri T Kurata A. An adolescent with Behcet’s aortitis mimicking infective endocarditis: a case report. Eur Heart J Case Rep. (2021) 5(10):ytab315. 10.1093/ehjcr/ytab315 34622128 PMC8493009

[27] Zuhlke L Karthikeyan G Engel ME Rangarajan S Mackie P Cupido-Katya Mauff B Clinical outcomes in 3343 children and adults with rheumatic heart disease from 14 low- and middle-income countries: two-year follow-up of the global rheumatic heart disease registry (the REMEDY study). Circulation. (2016) 134(19):1456–66. 10.1161/CIRCULATIONAHA.116.024769 27702773

[28] Coffey S Roberts-Thomson R Brown A Carapetis J Chen M Enriquez-Sarano M Global epidemiology of valvular heart disease. Nat Rev Cardiol. (2021) 18(12):853–64. 10.1038/s41569-021-00570-z 34172950

[29] Remenyi B ElGuindy A Smith SC Jr. Yacoub M Holmes DR Jr. Valvular aspects of rheumatic heart disease. Lancet. (2016) 387(10025):1335–46. 10.1016/S0140-6736(16)00547-X 27025439

[30] Summerhill VI Moschetta D Orekhov AN Poggio P Myasoedova VA. Sex-specific features of calcific aortic valve disease. Int J Mol Sci. (2020) 21(16):5620. 10.3390/ijms21165620 32781508 PMC7460640

[31] Lindman BR Clavel MA Mathieu P Iung B Lancellotti P Otto CM Calcific aortic stenosis. Nat Rev Dis Primers. (2016) 2:16006. 10.1038/nrdp.2016.6 27188578 PMC5127286

[32] Jeong DS Kim KH Kim JS Ahn H. Long-term experience of surgical treatment for aortic regurgitation attributable to Behcet’s disease. Ann Thorac Surg. (2009) 87(6):1775–82. 10.1016/j.athoracsur.2009.03.008 19463593

[33] Liang MY Yao JP Zhang X Wang ZP. Surgical technique for aortic regurgitation attributable to Behcet’s disease: modified aortic valve replacement with reinforcement of the aortic wall. Eur J Cardiothorac Surg. (2012) 41(3):647–8. 10.1093/ejcts/ezr025 22345185

[34] Ma WG Zheng J Zhu JM Liu YM Li M Sun LZ. Aortic regurgitation caused by Behcet’s disease: surgical experience during an 11-year period. J Card Surg. (2012) 27(1):39–44. 10.1111/j.1540-8191.2011.01392.x 22321112

[35] Lee I Park S Hwang I Kim MJ Nah SS Yoo B Cardiac Behcet disease presenting as aortic valvulitis/aortitis or right heart inflammatory mass: a clinicopathologic study of 12 cases. Am J Surg Pathol. (2008) 32(3):390–8. 10.1097/PAS.0b013e31814b23da 18300812

[36] Vahanian A Beyersdorf F Praz F Milojevic M Baldus S Bauersachs J 2021 ESC/EACTS guidelines for the management of valvular heart disease. Eur Heart J. (2022) 43(7):561–632. 10.1093/eurheartj/ehab395 34453165

[37] Flint N Wunderlich NC Shmueli H Ben-Zekry S Siegel RJ Beigel R. Aortic regurgitation. Curr Cardiol Rep. (2019) 21(7):65. 10.1007/s11886-019-1144-6 31161305

[38] Li X Wen X Xu J Lin Q Liu L. Prognostic analysis of Behcet’s disease with aortic regurgitation or involvement. Neth Heart J. (2022) 30(3):172–80. 10.1007/s12471-021-01567-6 33877589 PMC8881513

[39] Ahn JK Kim H Lee J Park PW Jeon CH Koh EM Treatment outcomes in patients with non-infectious aortic valvulitis undergoing aortic valve replacement: implication for the treatment of aortic valve involvement in Behcet’s disease. Rheumatol Int. (2009) 29(11):1391–3. 10.1007/s00296-009-0862-2 19169881

[40] Sun L Liu J Jin X Wang Z Li L Bai W Perioperative management with biologics on severe aortic valve regurgitation caused by Behcet syndrome: the experience from a single center. Ther Adv Chronic Dis. (2021) 12:20406223211026753. 10.1177/20406223211026753 34221307 PMC8221692

[41] Tang Y Xu Z Liao Z Xu J. Supraannular aortic replacement for severe valve detachment attributable to Behcet’s disease. Ann Thorac Surg. (2012) 94(2):e55–7. 10.1016/j.athoracsur.2012.05.060 22818338

[42] Yoshikawa K Hori H Fukunaga S Tayama E Aoyagi S. Aortic root replacement in Behcet disease. Asian Cardiovasc Thorac Ann. (2007) 15(6):521–3. 10.1177/021849230701500616 18042781

[43] Tsunekawa T Ogino H Matsuda H Minatoya K Sasaki H Kobayashi J Composite valve graft replacement of the aortic root: twenty-seven years of experience at one Japanese center. Ann Thorac Surg. (2008) 86(5):1510–7. 10.1016/j.athoracsur.2008.07.051 19049741

[44] Liang L Lai-chun S Hong-yan X Ming WBX Ji-hui F Xiao-yong L Clinical results of “chimney” Bentall procedure in patients with Behcet’s disease. Chin J Cardiovasc Res. (2022) 20:706–10. 10.3969/j.issn.1672-5301.2022.08.007

[45] Ogino H. Surgical strategy for refractory aortitis. Gen Thorac Cardiovasc Surg. (2019) 67(1):25–31. 10.1007/s11748-018-0885-2 29404904

[46] Chen LW Wu XJ Cao H Dai XF. Valved conduit attached to left ventricular outflow tract for valve detachment in Behcet’s disease. Ann Thorac Surg. (2017) 103(3):e301–3. 10.1016/j.athoracsur.2016.09.063 28219578

[47] Jiang JB Liu XB Gao F Fan JJ Lin XP Pu ZX A case report of transcatheter aortic valve replacement for severe aortic regurgitation in a patient with Behcet disease. Chin J Cardiol. (2021) 49(1):71–3. 10.3760/cma.j.cn112148-20200225-00127 33429490

[48] Makkar RR Thourani VH Mack MJ Kodali SK Kapadia S Webb JG Five-year outcomes of transcatheter or surgical aortic-valve replacement. N Engl J Med. (2020) 382(9):799–809. 10.1056/NEJMoa1910555 31995682

[49] Roy DA Schaefer U Guetta V Hildick-Smith D Mollmann H Dumonteil N Transcatheter aortic valve implantation for pure severe native aortic valve regurgitation. J Am Coll Cardiol. (2013) 61(15):1577–84. 10.1016/j.jacc.2013.01.018 23433565

[50] Yoon SH Schmidt T Bleiziffer S Schofer N Fiorina C Munoz-Garcia AJ Transcatheter aortic valve replacement in pure native aortic valve regurgitation. J Am Coll Cardiol. (2017) 70(22):2752–63. 10.1016/j.jacc.2017.10.006 29191323

[51] Avvedimento M Tang GHL. Transcatheter aortic valve replacement (TAVR): recent updates. Prog Cardiovasc Dis. (2021) 69:73–83. 10.1016/j.pcad.2021.11.003 34800439

[52] Coniglio AC Patel CB Kittleson M Schlendorf K Schroder JN DeVore AD. Innovations in heart transplantation: a review. J Card Fail. (2022) 28(3):467–76. 10.1016/j.cardfail.2021.10.011 34752907

[53] de Jonge N Kirkels JH Klopping C Lahpor JR Caliskan K Maat AP Guidelines for heart transplantation. Neth Heart J. (2008) 16(3):79–87. 10.1007/BF03086123 18345330 PMC2266869

[54] Hollander SA Yasnovsky JR Reinhartz O Chan F Sandborg C Hunt S Behcet’s disease and heart transplantation: a word of caution. J Heart Lung Transplant. (2010) 29(11):1306–8. 10.1016/j.healun.2010.07.010 20822920

[55] Guo X Tian Z Liu Y Li M Zeng X Fang Q. Preoperative immunosuppressive therapy reduces paravalvular leakage after aortic valve surgery in patients with aortic regurgitation attributable to Behcet’s disease. Clin Exp Rheumatol. (2016) 34(6 Suppl 102):S26–33. 26005767

[56] Karadag O Bolek EC. Management of Behcet’s syndrome. Rheumatology (Oxford). (2020) 59(Suppl 3):iii108–17. 10.1093/rheumatology/keaa086 32348509

[57] Choi HM Kim HK Park SJ Lee HJ Yoon YE Park JB Predictors of paravalvular aortic regurgitation after surgery for Behcet’s disease-related severe aortic regurgitation. Orphanet J Rare Dis. (2019) 14(1):132. 10.1186/s13023-019-1083-8 31182113 PMC6558675

